# Tumor survivors’ musculoskeletal health assessment: advances in imaging biomarkers for cancer treatment-related sequelae

**DOI:** 10.1186/s13018-026-07090-x

**Published:** 2026-07-02

**Authors:** Jingxin He, Huichun Xiang, Shufen Xiao, Xingshun Zhou

**Affiliations:** 1 The First People’s Hospital of Zhaotong City, Yunnan Province, Zhaotong, 657000 Yunnan China; 2 Huaihua Traditional Chinese Medicine Hospital, Huaihua, 418000 Hunan China; 3 No. 926 Hospital, Joint Logistics Support Force of PLA, Kaiyuan, 661699 Yunnan China

**Keywords:** Tumor survivors, Musculoskeletal health, Cancer treatment-related sequelae, Imaging biomarkers, Sarcopenia, Osteoporosis, Radiologic assessment

## Abstract

With significant advancements in cancer treatment, the population of cancer survivors has grown substantially, bringing long-term health challenges to the forefront of clinical concern. Among these, cancer treatment-related musculoskeletal sequelae critically impact survivors’ quality of life, functional independence, and overall survival. Musculoskeletal health deterioration—including bone density loss, sarcopenia, myosteatosis, osteonecrosis, and muscle damage—represents a complex clinical issue that requires precise evaluation and monitoring. Imaging technologies have emerged as essential non-invasive tools for identifying, quantifying, and tracking these late effects. This review systematically examines the current landscape of imaging biomarkers used to assess musculoskeletal health in cancer survivors, emphasizing their associations with common cancer therapies such as radiotherapy, chemotherapy, endocrine therapy, and targeted and immunotherapies. We explore the application and recent advances of various imaging modalities—including dual-energy X-ray absorptiometry (DXA), quantitative computed tomography (QCT), magnetic resonance imaging (MRI), positron emission tomography/computed tomography (PET/CT), and ultrasound imaging—in detecting and characterizing treatment-induced musculoskeletal alterations. Moreover, the review highlights the translational potential of these imaging biomarkers in clinical practice, aiming to enhance early detection, guide personalized interventions, and improve long-term outcomes for cancer survivors. By consolidating current evidence and identifying existing gaps, this article provides a comprehensive overview of the role of imaging biomarkers in addressing the critical issue of musculoskeletal health in the growing population of cancer survivors.

## Introduction

This review article addresses the pressing issue of musculoskeletal complications faced by cancer survivors, particularly focusing on the implications of cancer treatment-related sequelae. Cancer survivors often encounter a myriad of long-term health challenges, among which complications affecting the musculoskeletal system are notably significant. Treatments such as chemotherapy, radiation therapy and hormonal therapies while effective in combating cancer frequently exert detrimental effects on bone and muscle tissues. These adverse effects can lead to conditions such as treatment-induced osteoporosis, increased fracture risk, sarcopenia associated with cancer, and other musculoskeletal impairments that profoundly impact cancer survivors’ quality of life.

Musculoskeletal complications can manifest as treatment-induced bone loss and muscle weakness, which are critical factors influencing the overall health and survivorship of cancer survivors. Osteoporosis, characterized by decreased bone density and increased fragility, is a common outcome of various cancer therapies, particularly in populations such as breast and prostate cancer survivors undergoing androgen deprivation therapy. The interplay between cancer treatment and musculoskeletal health highlights the need for comprehensive monitoring and management strategies to mitigate these complications. Moreover, the association between musculoskeletal health and treatment tolerability emphasizes the importance of addressing these issues early in the survivorship continuum to enhance treatment outcomes and quality of life.

Given the importance of assessing musculoskeletal complications, recent advancements in imaging technologies have provided new avenues for the evaluation and monitoring of musculoskeletal health in cancer survivors. Imaging biomarkers derived from advanced modalities such as MRI and CT scans offer objective, quantifiable metrics aiding early detection of musculoskeletal complications. These imaging techniques facilitate a better understanding of the pathophysiological changes occurring in bone and muscle tissues post-treatment, allowing for timely interventions that can improve functional outcomes and reduce the risk of further complications.

In light of these challenges, this review aims to systematically explore the current landscape of imaging-based biomarkers related to cancer treatment-induced musculoskeletal complications. By analyzing the physiological underpinnings of these changes, evaluating the advantages and limitations of various imaging modalities, and exploring potential future research directions, this review seeks to provide a comprehensive overview that can inform clinical practices and improve long-term health management for cancer survivors. The insights gained from this analysis could be instrumental in developing targeted interventions that enhance musculoskeletal health and long-term survivorship outcomes in this vulnerable population.

### Literature Search Strategy

This systematic narrative review was conducted following standard narrative review specifications.

1. Databases

PubMed, Web of Science, Cochrane Library were retrieved for peer-reviewed full-text articles.

2. Search Terms

English keywords: tumor survivor, cancer treatment-related musculoskeletal sequelae, imaging biomarker, sarcopenia, osteoporosis, DXA, QCT, MRI, PET/CT, shear wave elastography, artificial intelligence, myosteatosis.

3. Time Range

All literature published from January 2010 to September 2025 was screened, with priority given to clinical cohort studies, translational imaging research, and systematic reviews published in the last 5 years.

4. Inclusion Criteria

(1) Clinical or preclinical studies investigating bone/muscle damage induced by chemotherapy, radiotherapy, endocrine therapy, targeted therapy or immunotherapy; (2) Research adopting quantitative imaging parameters to quantify musculoskeletal structure, metabolism or function; (3) Full-text articles published in English or Chinese with complete original data; (4) Studies enrolling tumor survivors as core research objects.

5. Exclusion Criteria

(1) Case reports, conference abstracts, grey literature without peer review; (2) Imaging studies focusing on primary musculoskeletal diseases without tumor treatment background; (3) Reviews lacking quantitative imaging biomarker analysis; (4) Animal experiments without translational clinical value.

6. Screening Process

Two authors independently screened titles and abstracts for preliminary filtering, followed by full-text re-evaluation. Disagreements were resolved via discussion with the corresponding author. Literature information including imaging modalities, quantitative biomarkers, pathological mechanisms and clinical endpoints was extracted for integrated analysis.

## Cancer treatment-related musculoskeletal sequelae: pathophysiological basis and clinical significance

### Specific mechanisms of treatment modalities on bone health

Cancer therapies, including chemotherapy, radiotherapy, endocrine therapy, and targeted agents, exert distinct and multifactorial effects on bone health through diverse pathophysiological mechanisms. Chemotherapy-induced bone loss arises from direct cytotoxicity to bone-forming osteoblasts and an imbalance in osteoclast activity. Agents such as doxorubicin have been shown to accelerate bone resorption by activating inflammasome pathways, notably AIM2 and NLRP3, leading to inflammatory cell death and cytokine release, which promote osteoclastogenesis [1]. Methotrexate, commonly used in pediatric oncology, impairs bone growth and increases marrow adiposity by upregulating Notch2 signaling. Notch2 signaling modulates osteoclast differentiation and suppresses osteogenesis [2]. Additionally, chemotherapy-induced bone loss involves mechanisms beyond estrogen and/or androgen deprivation, with cellular senescence and its associated secretory phenotype (SASP) playing a major role in promoting bone loss [3]. The long-term impact of chemotherapy on bone is also mediated by cellular senescence, which drives bone resorption. However, senolytic agents targeting senescent cells have not consistently prevented chemotherapy-related bone loss, indicating complex underlying mechanisms [4].

Radiotherapy causes long-term skeletal damage primarily through microvascular injury, osteocyte death, and fibrosis, culminating in radiogenic bone necrosis conditions such as osteoradionecrosis of the jaw and femoral head necrosis. The damage to bone vasculature and cellular components impairs remodeling and repair, increasing fracture risk [5, 6].Radiation-induced marrow adipose tissue expansion is linked to alterations in hypoxia-inducible factor (HIF) signaling, with aP2-expressing stromal cells regulating marrow adiposity and bone integrity post-irradiation. Preclinical animal experiments have confirmed that cranial irradiation can trigger distant skeletal muscle atrophy and persistent fatigue in rats, mediated by systemic inflammatory cascades [7]. While salivary gland studies confirm radiation-induced mitochondrial dysfunction and impaired energy metabolism [8], direct evidence verifying that radiotherapy disrupts mitochondrial energy metabolism within skeletal muscle tissue remains absent and requires further mechanistic exploration.

Endocrine therapies represented by aromatase inhibitors and androgen deprivation therapy induce bone loss mainly through systemic sex hormone withdrawal, which disrupts bone turnover balance and increases fracture risk. Clinical cohort data confirm that aromatase inhibitors frequently trigger extraosseous musculoskeletal adverse events including carpal tunnel syndrome and stenosing tenosynovitis, which may lead to premature treatment discontinuation if unrecognized [9]. Existing literature focuses on clinical adverse event phenotypes, while molecular pathways linking hormone deprivation to osteoclast overactivation need more mechanistic verification.

**Fig. 1:**
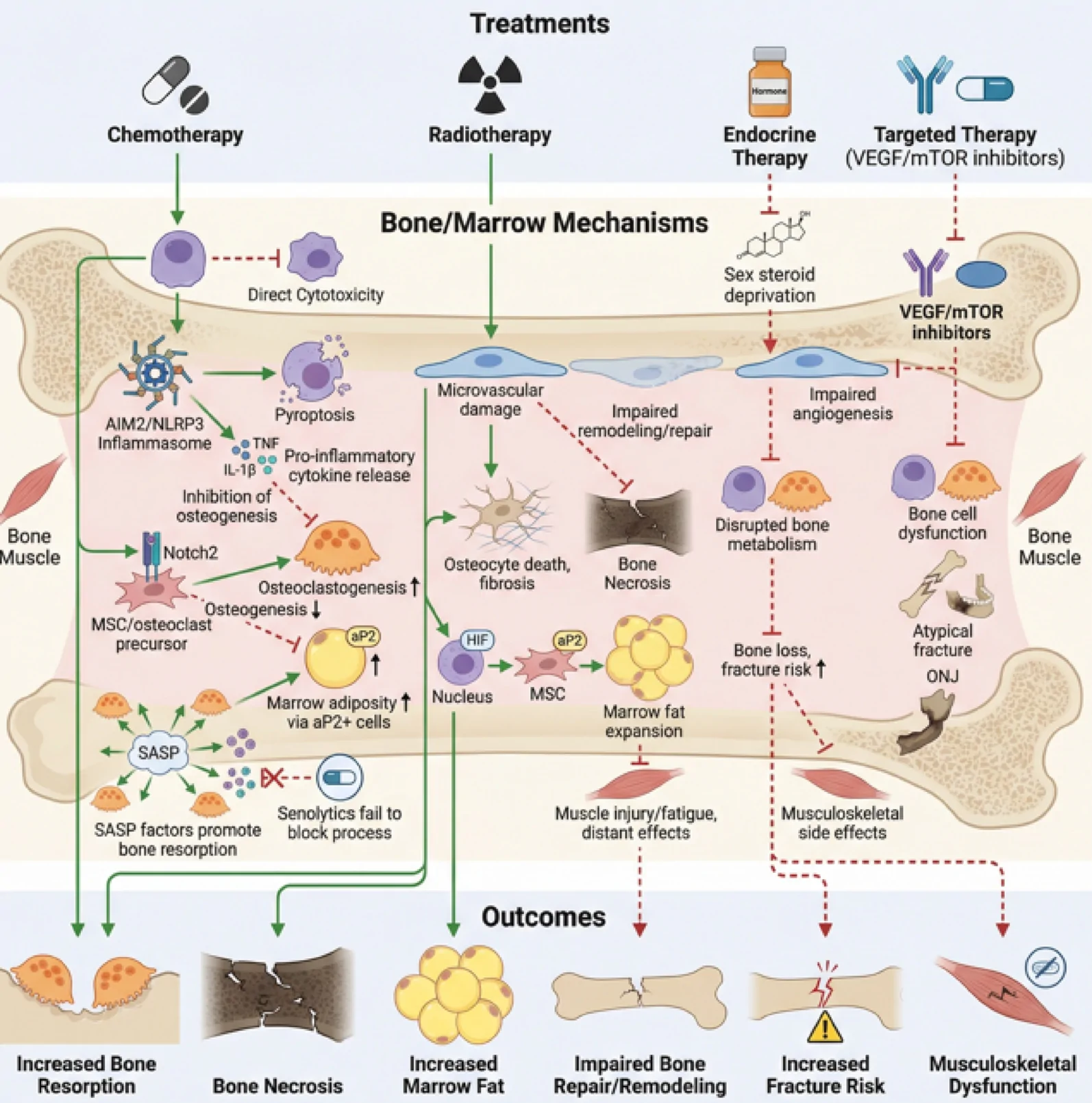
Specific mechanisms of treatment modalities on bone health

Targeted therapies, including vascular endothelial growth factor (VEGF) and mTOR inhibitors, contribute to skeletal complications by interfering with angiogenesis and bone remodeling signaling pathways. These agents have been associated with atypical fractures and osteonecrosis of the jaw (ONJ), potentially through vascular compromise and direct effects on osteoclasts and osteoblasts. The multifaceted impact of these therapies underscores the need for vigilant bone health monitoring and tailored interventions (Fig. 1).

### Pathogenesis of cancer treatment-related muscle wasting (CTRM)

Cancer treatment-related muscle wasting (CTRM) develops under the combined action of systemic inflammation, metabolic disturbance, mitochondrial injury, neuromuscular junction dysfunction and anabolic resistance, though the causal hierarchy of each pathway remains incompletely clarified in tumor survivor cohorts [10].Chemotherapy and immunotherapy can trigger muscle protein degradation. Chronic systemic inflammation and elevated pro-inflammatory cytokines are well-established drivers of muscle atrophy in general cancer cachexia, which activate ubiquitin–proteasome and autophagy proteolytic pathways [10, 11].however, tumor treatment-specific experimental evidence linking anti-tumor drugs directly to these proteolytic cascades is still insufficient. Mitochondrial toxicity is hypothesized to mediate myofiber atrophy: anthracyclines are suspected to suppress ATP synthesis and amplify oxidative stress, yet direct cellular experimental data confirming doxorubicin-induced mitochondrial damage in skeletal muscle remain limited [10, 12].

Peripheral neuropathy induced by chemotherapeutic agents such as taxanes and platinum compounds may contribute to muscle dysfunction; however, the cited reference does not specifically address disruption of neuromuscular junction integrity or muscle innervation leading to disuse atrophy and functional decline. Fatigue and reduced physical activity during treatment further exacerbate muscle loss through disuse mechanisms linked to neuromuscular impairment. Anabolic resistance characterized by impaired insulin-like growth factor-1 (IGF-1) signaling and reduced muscle protein synthesis response to amino acids and exercise has been proposed in CTRM; however, this is not directly supported by the cited references [3, 10].

Emerging evidence highlights the role of senescence-associated secretory phenotype (SASP) in promoting inflammation and bone loss; however, the cited reference does not address alterations in bone-muscle crosstalk or muscle wasting in cancer survivors [3].The interplay between systemic inflammation, mitochondrial dysfunction, and impaired neuromuscular junction signaling creates a vicious cycle that sustains muscle catabolism and functional impairment.

In summary, cancer therapies induce bone and muscle toxicities through direct cellular damage, hormonal alterations, inflammation, and metabolic dysregulation. These sequelae have profound clinical implications, including increased fracture risk, reduced mobility, and diminished quality of life, necessitating comprehensive survivorship care with multidisciplinary approaches for prevention, early detection, and management.

### Imaging biomarkers for assessing bone health

#### Quantitative markers of bone density and microstructure

Accurate quantification of bone density and microstructural parameters is essential to evaluate bone strength and predict fracture risk. Dual-energy X-ray absorptiometry (DXA) remains the clinical gold standard for measuring areal bone mineral density (aBMD) at the lumbar spine and hip, with T-score and Z-score as core diagnostic criteria for osteoporosis and fracture risk stratification. Nevertheless, DXA has inherent limitations: two-dimensional projection images cannot distinguish cortical and trabecular bone compartments or resolve fine microarchitecture, and measurements are easily confounded by bone size, spinal degenerative lesions, vascular calcification and soft tissue artifacts, as comprehensively summarized in previous radiological methodological studies [13–15].

Quantitative computed tomography (QCT) and its high-resolution peripheral variant (HR-pQCT) provide volumetric bone mineral density (vBMD) measurements and detailed three-dimensional microstructural parameters, such as trabecular number, thickness, separation, cortical thickness, and porosity. These parameters allow a more precise evaluation of bone strength and a better discrimination of cortical versus trabecular bone changes, which is especially valuable in early detection of microstructural deterioration during treatment or disease progression [16–18]. For example, HR-pQCT studies in astronauts have demonstrated incomplete recovery of trabecular microarchitecture and bone strength after prolonged spaceflight, highlighting the sensitivity of these measures to subtle bone changes. Similarly, HR-pQCT reveals alterations in bone microstructure not captured by DXA; however, the cited references do not specifically demonstrate region-specific alterations in osteoarthritis and osteoporosis [19, 20].

Advanced MRI modalities including chemical shift encoding-based MRI (CSE-MRI) and magnetic resonance spectroscopy (MRS) quantify bone marrow fat fraction (FF), a novel surrogate marker of overall bone quality. Preclinical studies propose that elevated marrow adiposity compromises bone strength and increases fracture susceptibility, yet prospective clinical data directly correlating marrow FF with fracture events in tumor survivors are lacking [16, 21, 22].Advanced MRI techniques such as ultrashort echo time (UTE) MRI can also estimate cortical porosity and bound water content, providing insights into bone matrix organization and hydration that complement density and microstructure assessments [16, 23].

Emerging imaging modalities such as photoacoustic imaging have shown promise in characterizing bone chemical composition and microstructure noninvasively, potentially offering radiation-free alternatives for bone health assessment [24–26].

In summary, while DXA-derived aBMD remains central in clinical practice, quantitative CT and MRI-based biomarkers provide more comprehensive and sensitive assessments of bone density, microarchitecture, and marrow composition. These advanced imaging biomarkers are critical for early diagnosis, monitoring treatment response, and improving fracture risk prediction, especially in populations with complex bone disorders or treatment-related bone loss.

#### Bone turnover and metabolic activity biomarkers

Bone turnover represents dynamic remodeling processes that determine skeletal integrity; functional imaging biomarkers reflect metabolic activity beyond static structural density measurements. Nuclear medicine modalities including bone scintigraphy and PET provide unique insights into bone remodeling physiology.

^18^F-sodium fluoride PET/CT (^18^F-NaF PET/CT) sensitively captures regional bone metabolism and blood perfusion by targeting sites of active bone turnover, with standardized uptake value (SUV) as the core quantitative readout. In osteoarthritis cohorts, ^18^F-NaF PET/MRI successfully identifies bone remodeling patterns correlated with disease severity and supports early lesion detection and therapeutic response monitoring. The technique is widely applied to evaluate bone metastases and metabolic bone diseases [27], yet its capacity to detect pre-structural metabolic abnormalities induced by radiotherapy or systemic anti-tumor drugs has not been validated in prospective longitudinal cancer survivor cohorts [28–30].

Radiomic texture analysis applied to conventional CT images is an emerging approach to extract quantitative features that characterize bone heterogeneity and microarchitecture. Texture features derived from gray-level co-occurrence matrices and other statistical models have shown promise in various imaging analyses; however, the cited references do not specifically support their use in predicting pathological fracture risk, differentiating benign from malignant bone lesions, or assessing treatment response [31, 32]. These radiomic biomarkers can capture subtle variations in bone texture that correlate with underlying biological changes, enabling earlier and more precise risk stratification.

Hybrid imaging modalities such as SPECT/CT and PET/MRI integrate metabolic and anatomical information, enhancing diagnostic accuracy in various clinical contexts; however, the cited reference does not specifically support their application in musculoskeletal disorders including infection, inflammation, and oncology [33]. The combination of biochemical markers of bone turnover with imaging biomarkers further refines the assessment of bone metabolic activity and remodeling dynamics[34, 35].

**Fig. 2:**
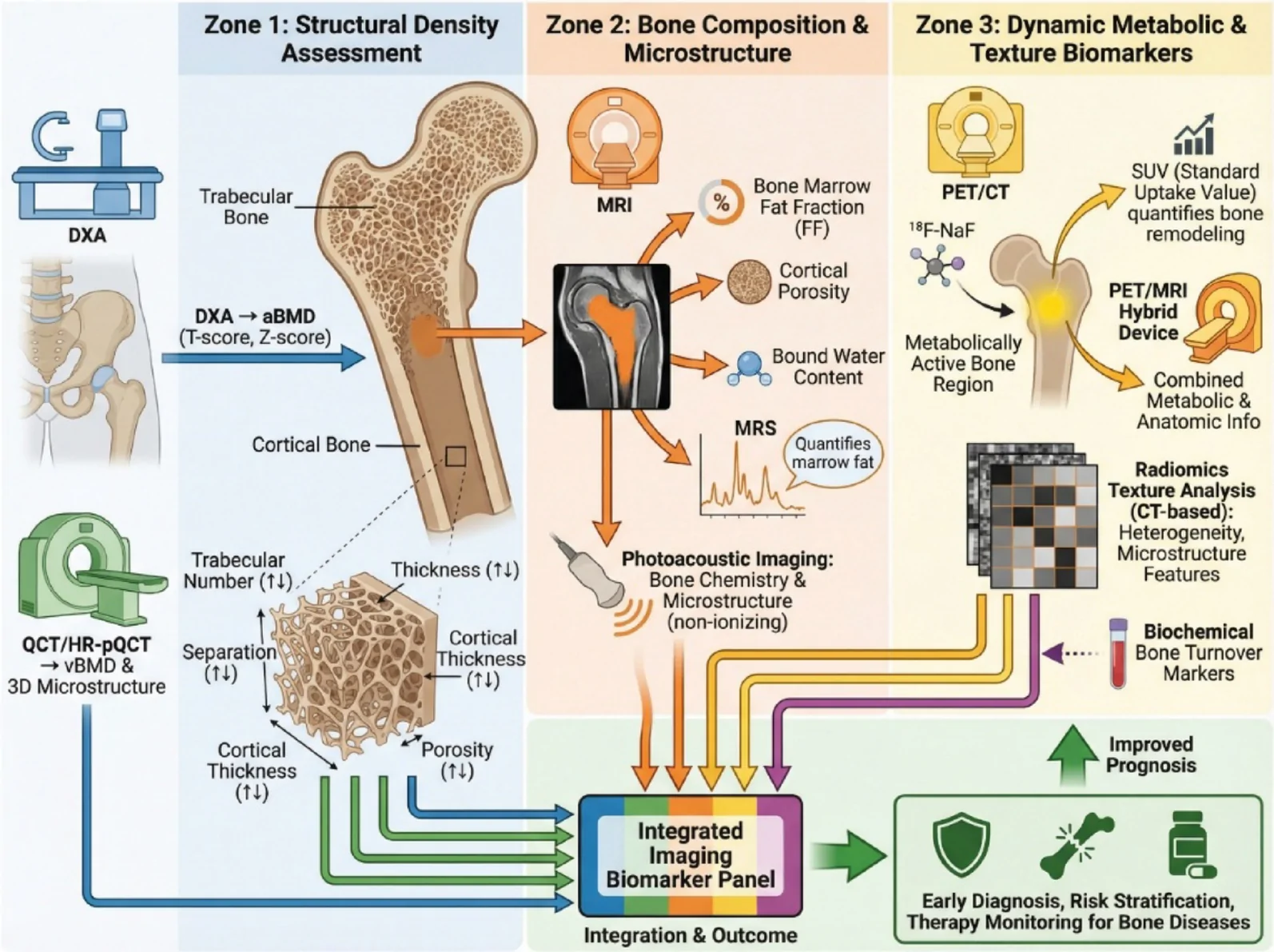
Imaging biomarkers for assessing bone health

In summary, nuclear medicine imaging biomarkers such as 18F-NaF PET/CT SUV and CT-based radiomic texture features provide critical functional and metabolic information about bone turnover and remodeling. These biomarkers complement structural imaging and biochemical assays, facilitating early detection of bone pathology, monitoring of treatment effects, and improved prediction of fracture risk and disease progression (Fig. 2).

## Imaging biomarkers for assessing muscle health

### Muscle quantity and morphological biomarkers

#### CT and MRI cross-sectional area (CSA) and volume measurements

CTand MRIhave become gold-standard modalities for quantifying skeletal muscle mass, particularly through cross-sectional area (CSA) and volumetric measurements at the third lumbar vertebra (L3) level. The skeletal muscle index (SMI), defined as the muscle CSA normalized by height squared (cm^2^/m^2^), is widely used as a diagnostic criterion for sarcopenia and muscle wasting. Automated deep learning pipelines have been developed to segment skeletal muscle at L3 on CT images, enabling high-throughput and reproducible measurements in large cohorts [36]. These CT-derived muscle metrics strongly correlate with clinical outcomes, including postoperative complications, chemotherapy toxicity, and overall survival. For example, higher SMI is associated with decreased risk of cardiac dysrhythmias and epilepsy, while decubitus ulcers are related to skeletal muscle density (SMD) rather than SMI, highlighting the clinical relevance of muscle quantity and quality assessment [36]. MRI, similarly, offers high soft-tissue contrast and can provide volumetric muscle data beyond single-slice CSA. Multiparametric MRI enables detailed characterization of muscle size, fatty infiltration, and microstructure [37]. The standard protocol involves segmenting the paraspinal and abdominal muscles at L3, CSA, and normalizing by height squared to yield skeletal muscle index (SMI); however, established cutoff values for sarcopenia diagnosis, particularly in epithelial ovarian cancer (EOC), have not been definitively defined [38]. This approach has been proposed for assessing biological aging using CT biomarkers; however, validation in various clinical populations including cancer patients and older adults, and its incorporation into frailty and biological aging assessments, remain to be established [39, 40].Recent advances in convolutional neural networks allow for data-efficient muscle segmentation on CT and MRI, achieving human-level accuracy with reduced annotation requirements [41]. Overall, CT- and MRI-based CSA and volume measurements at L3 provide robust, reproducible imaging biomarkers for muscle quantity and morphology, with strong associations to clinical outcomes.

### Quantitative assessment of muscle fat infiltration (Myosteatosis)

**Fig. 3:**
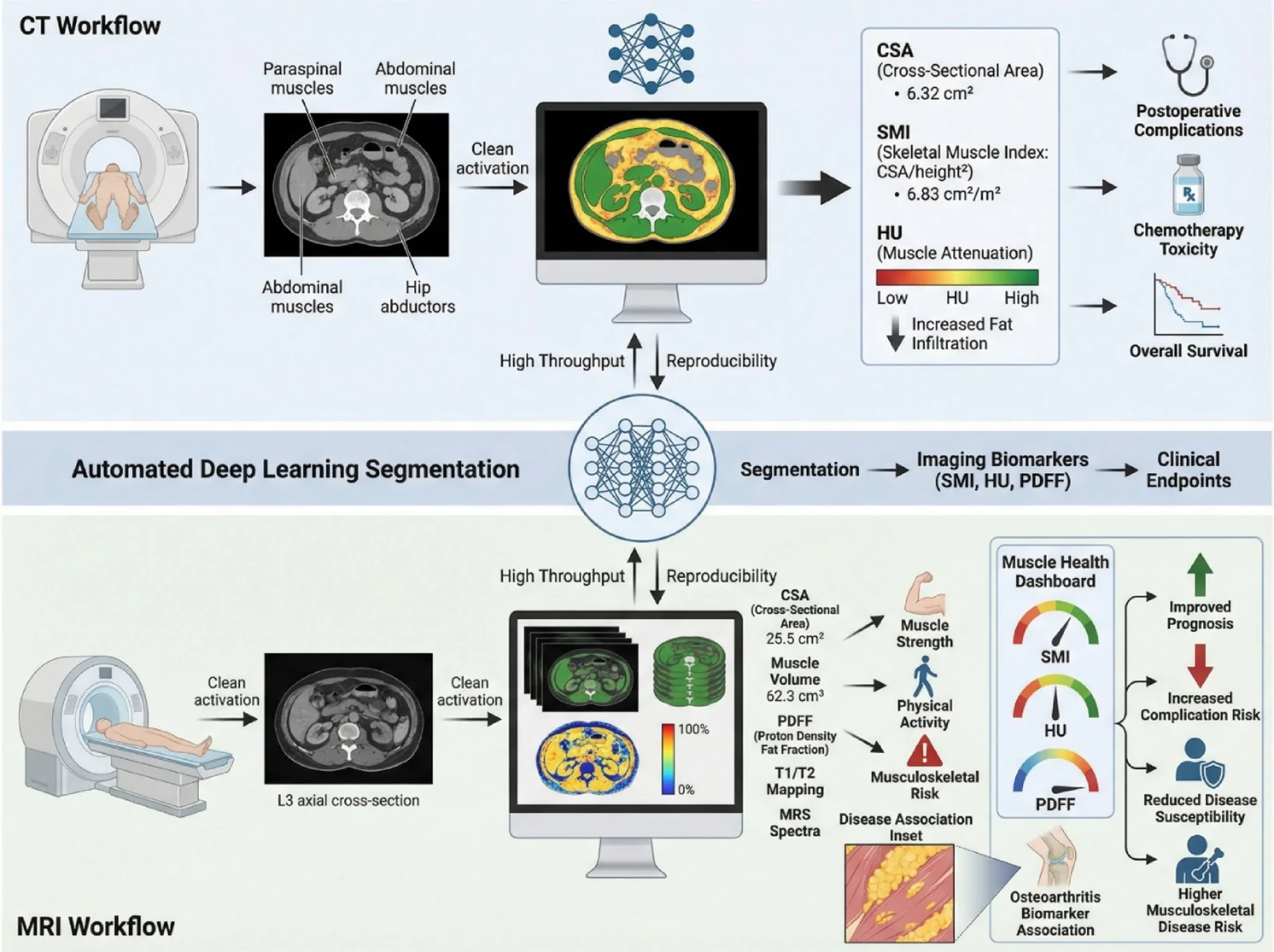
Muscle quantity and morphological biomarkers

Muscle fat infiltration, or myosteatosis, is characterized by ectopic fat deposition within skeletal muscle, adversely affecting muscle quality and function. CT attenuation values, expressed in Hounsfield units (HU), serve as a surrogate for muscle fat content, with lower muscle HU indicating higher fat infiltration [42] Typically, muscle attenuation thresholds range from − 29 to + 150 HU, with lower mean HU values correlating with decreased muscle strength, mobility, and increased morbidity. MRI-based techniques, such as proton density fat fraction (PDFF) mapping using chemical-shift encoding, provide direct quantification of intramuscular fat with high sensitivity and specificity [42]. PDFF mapping can be complemented by T1-T2 mapping and magnetic resonance spectroscopy (MRS) to assess muscle composition and metabolic status.Ultrasound elastography and echo intensity measurements also offer bedside assessment of muscle fat infiltration, although these techniques require further validation.Myosteatosis is an independent predictor of adverse outcomes in aging, cancer cachexia, and chronic diseases, and is distinct from sarcopenia, though they often coexist [42]. Quantitative CT and MRI assessments of muscle fat infiltration have been linked to frailty in diverse populations; however, associations with postoperative complications and survival are not clearly supported by the cited references [36, 40]. Automated segmentation and fat quantification methods have been developed to enable precise measurement of muscle fat fraction in specific muscle groups, such as the hip abductors, and to investigate their association with early osteoarthritis biomarkers [43].Collectively, imaging biomarkers of muscle fat infiltration derived from CT HU values and MRI PDFF mapping represent sensitive and clinically relevant measures of decline in muscle quality (Fig. 3).

### Muscle quality and functional imaging biomarkers

#### Ultrasound elastography and shear wave velocity

Shear wave elastography (SWE) quantifies shear wave velocity (SWV) to reflect muscle tissue stiffness, acting as a non-invasive marker of muscle quality. Novel real-time pulse-echo speed-of-sound (SoS) ultrasound accurately assesses deep muscle tissue, with SoS values significantly correlated with BMI, physical activity levels and sex differences in human subjects [44, 45]. Current SoS research focuses on anthropometric associations, while direct correlations between SWV and intramuscular fat, fibrosis, muscle strength or functional performance have not been systematically validated in human cohorts [44, 45]. Deep learning denoising algorithms improve SWV reconstruction under low signal-to-noise conditions, yet validation experiments are limited to tissue phantoms and ex vivo animal liver samples without fatty muscle tissue testing [46]. Frequency-domain cyclic shift algorithms further enhance SWV estimation stability [47]. SWE has been applied to quantify tendon stiffness in hyperlipidemia patients [48], while its diagnostic value for neuromuscular disorders and exercise-induced muscle hyperemia remains understudied [49, 50].A major limitation of ultrasound elastography is operator dependence; standardized acquisition and post-processing protocols to improve inter-operator reproducibility need further optimization [51, 52].

#### Diffusion tensor imaging (DTI) and magnetic resonance spectroscopy (MRS)

DTI extracts microstructural metrics including fractional anisotropy (FA) and apparent diffusion coefficient (ADC) to reflect skeletal muscle fiber integrity, orientation and anisotropy. DTI parameters correlate with muscle strength and pathological changes in inherited neuromuscular diseases, yet dedicated applications for cancer treatment-related muscle injury are rarely reported.MRS quantifies intramuscular metabolites such as creatine, choline and lipids to characterize energy metabolism and tissue composition, but its implementation in monitoring chemotherapy-induced myopathy remains exploratory [37, 53].Combined DTI-MRS multiparametric MRI provides comprehensive muscle quality profiling, yet most existing literature focuses on idiopathic inflammatory myopathies and muscular dystrophies rather than tumor survivor cohorts [53, 54].DTI’s potential to assess muscle remodeling in obesity and type 2 diabetes exists, but metabolite alterations associated with treatment-related muscle atrophy lack systematic MRS verification[55].

#### Dynamic contrast-enhanced mri and blood oxygen level dependent (BOLD) MRI

Dynamic contrast-enhanced (DCE) MRI evaluates perfusion and microvascular permeability in various tissues; however, the cited references do not specifically address muscle perfusion, microvascular permeability, or vascularization [54, 56].Blood oxygen level dependent (BOLD) MRI measures changes in deoxyhemoglobin concentration, reflecting tissue oxygenation and hemodynamics; however, the cited references do not specifically address muscle oxygenation or hemodynamics during rest and exercise. Functional MRI techniques have been applied in neuromuscular and metabolic disorders; however, the cited references do not specifically support their use in monitoring muscle response to exercise, detecting microvascular disease, or assessing treatment effects [54, 57]. DCE-MRI parameters such as Ktrans and kep have been studied in osteosarcoma treatment response; however, correlations with muscle vascular maturity are not reported in the cited reference [58]. BOLD MRI has been used in muscle imaging; The integration of DCE and BOLD MRI with other imaging biomarkers enhances understanding of muscle physiology and pathology beyond morphological assessment.

**Fig. 4:**
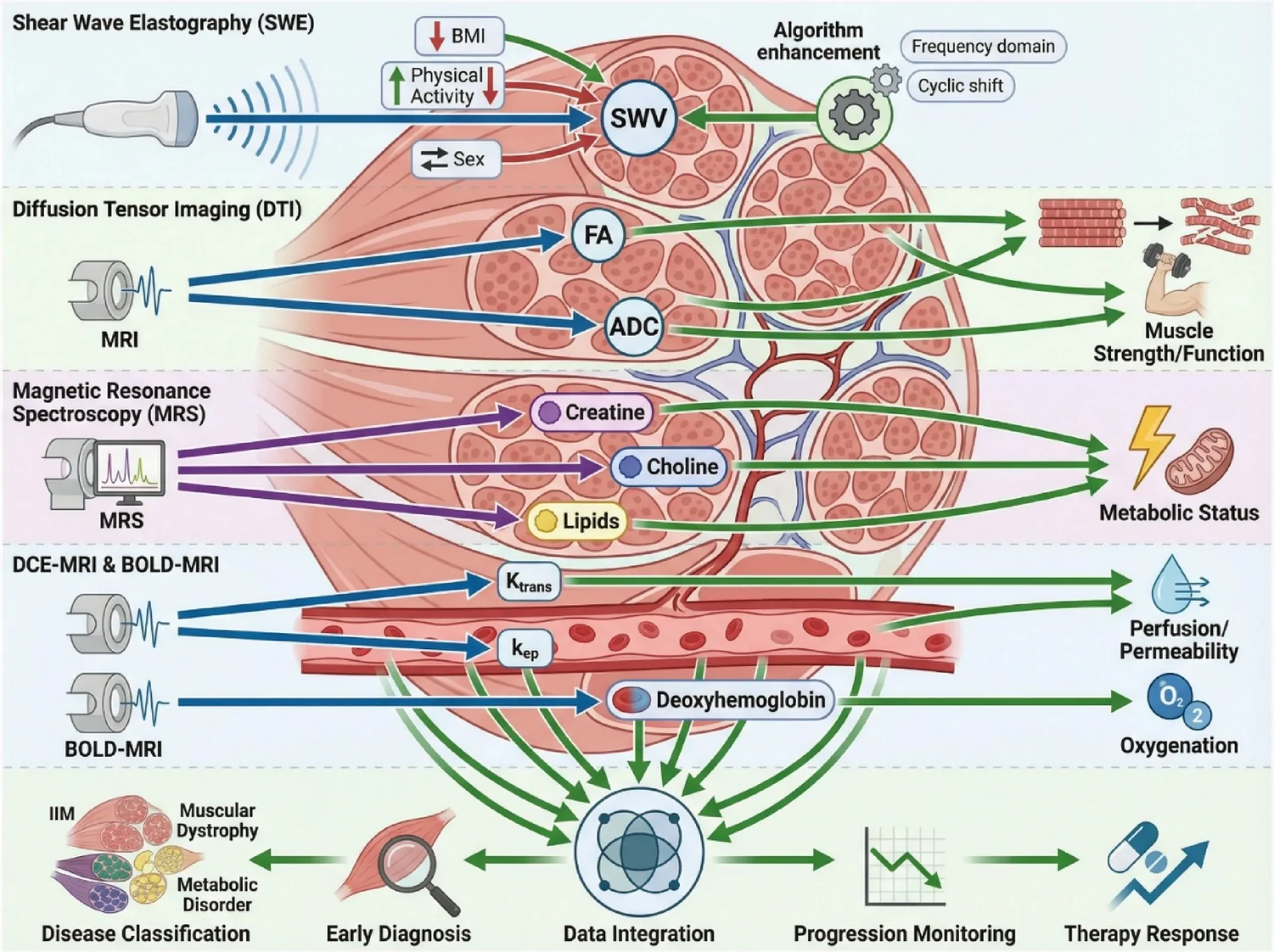
Muscle quality and functional imaging biomarkers

In summary, advanced imaging biomarkers encompassing ultrasound elastography, diffusion tensor imaging, magnetic resonance spectroscopy, and functional MRI techniques provide comprehensive quantitative and qualitative assessments of muscle quality and function. These biomarkers are increasingly recognized for their potential to improve diagnosis, monitor disease progression, and evaluate therapeutic interventions in muscle-related disorders (Fig. 4).

## Clinical applications, challenges, and future directions of imaging biomarkers

### Integrated applications in cancer survivor management

Imaging biomarkers have become indispensable tools in the comprehensive management of cancer survivors, particularly for assessing bone and muscle health, which are often compromised due to cancer treatments. The integration of quantitative imaging modalities such as dual-energy X-ray absorptiometry (DXA), quantitative computed tomography (QCT), and muscle indices derived from computed tomography (CT) allow for effective risk stratification and early warning of musculoskeletal complications. For instance, bone mineral density (BMD) measurements via DXA or QCT can identify survivors at high risk of osteoporosis or fractures before clinical manifestations, enabling timely intervention. CT-derived muscle indices, including skeletal muscle index (SMI) and muscle attenuation measured in Hounsfield units (HU), are important imaging biomarkers; however, the cited reference does not specifically link these indices to sarcopenia, muscle quality deterioration, functional decline, or treatment tolerance.Longitudinal imaging assessments can monitor treatment effects by quantifying changes in bone mineral density (BMD) and muscle mass or fat infiltration; however, the cited reference does not specifically address monitoring of bisphosphonates, denosumab, nutritional support, or exercise prescriptions [59]. Low skeletal muscle index (SMI) and reduced muscle Hounsfield unit (HU) values have been proposed as prognostic factors in cancer survivors [60].Multimodal imaging biomarkers combined with artificial intelligence (AI) algorithms have potential for data integration and interpretation; however, the cited reference primarily discusses diagnostic applications and does not specifically address personalized survivorship care, precise risk stratification, or tailored interventions [61].In summary, the integrated use of bone density and muscle imaging biomarkers in cancer survivorship management offers a powerful approach for early detection of musculoskeletal complications, treatment monitoring, and prognostic evaluation.

### Current challenges and need for standardization

Despite the promising clinical applications of imaging biomarkers in cancer survivorship, several challenges hinder their widespread adoption and standardization. Variability introduced by differences in imaging equipment, scanning protocols, and analysis software can lead to measurement inconsistencies and hinder comparability across centers; however, the cited reference does not specifically discuss these issues [59]. This variability underscores the urgent need to establish multicenter, large-sample reference values specific to cancer populations, as well as standardized operating procedures for image acquisition and analysis. Additionally, cost-effectiveness issues and accessibility challenges pose significant difficulties, especially when advanced imaging modalities such as whole-body MRI or positron emission tomography (PET) are considered for routine assessments. Balancing the precision of imaging biomarkers with resource constraints requires careful evaluation. Optimizing clinical utility while minimizing financial burden is important; cost-effectiveness analyses balancing diagnostic precision with medical resource allocation remain insufficient [60]. Furthermore, the clinical translation of imaging biomarkers necessitates rigorous validation through prospective, longitudinal studies that correlate imaging parameters with hard clinical endpoints such as fractures, severe disability—defined here as substantial loss of independence in daily activities—and mortality. Such validation is essential to establish causal relationships and to incorporate imaging biomarkers into clinical practice guidelines [60]. In addition, regulatory frameworks and quality assurance standards must evolve to accommodate the complexity of AI-enhanced imaging biomarkers, ensuring reliability and reproducibility in clinical settings [62]. Addressing these challenges through concerted efforts in standardization, cost–benefit analysis, and validation will be pivotal for the successful clinical integration of imaging biomarkers in cancer survivorship care.

### Integration of emerging technologies and artificial intelligence (AI)

The advent of emerging technologies, particularly artificial intelligence (AI), has revolutionized the field of imaging biomarker analysis, offering unprecedented opportunities for automation, accuracy, and personalized risk prediction. Deep learning algorithms have demonstrated capabilities in imaging data analysis; however, the cited reference does not specifically report on automated segmentation of skeletal muscle and bone regions or enhancements in analysis efficiency and reproducibility [61]. AI-based convolutional neural networks have potential for accurate delineation of muscle and bone boundaries and extraction of quantitative biomarkers.Multimodal data fusion approaches integrating imaging features with clinical and genomic data via machine learning models have potential for individualized risk assessment and intervention optimization [61]. These integrative models leverage the complementary information from diverse data sources to improve prognostic accuracy and support precision medicine in cancer survivorship. The application of foundation models—large-scale AI models pre-trained on extensive radiographic datasets—further enhances the robustness and biological relevance of imaging biomarkers, even in scenarios with limited training samples [63]. Looking ahead, the fusion of AI-driven automated image analysis with multimodal data integration holds great potential to transform survivorship care by enabling early detection of musculoskeletal complications, dynamic monitoring of treatment responses, and personalized therapeutic strategies. Successful implementation of advanced imaging technologies requires addressing challenges such as data standardization, interpretability, and clinical validation [61].

In conclusion, cancer treatment-related musculoskeletal sequelae represent a pivotal challenge in the long-term prognosis of cancer survivors, necessitating precise and objective assessment tools to guide clinical management. Imaging biomarkers have emerged as indispensable instruments in this domain, offering quantitative insights that transcend traditional clinical evaluation. The integration of bone imaging markers—such as DXA-derived areal bone mineral density (aBMD), QCT-based volumetric bone mineral density (vBMD), and high-resolution peripheral quantitative computed tomography (HR-pQCT) parameters of bone microarchitecture—with muscle imaging indices like the L3-level CT skeletal muscle index and muscle attenuation values (HU) has demonstrated robust clinical relevance and prognostic significance. These modalities enable clinicians and researchers to delineate the extent of musculoskeletal compromise with greater precision, facilitating risk stratification and tailored interventions.

Moreover, the advent of functional imaging techniques—including ^18F-sodium fluoride (NaF) PET for bone metabolism, MRI-based fat quantification, elastography ultrasound, and diffusion tensor imaging (DTI)—provides complementary metabolic, compositional, and functional data that enrich our understanding of the pathophysiological underpinnings of cancer treatment-induced bone and muscle alterations. These advanced modalities represent a promising frontier, potentially unveiling early subclinical changes and mechanistic pathways that conventional imaging might overlook. Their incorporation into research and clinical practice is anticipated to refine diagnostic accuracy and therapeutic monitoring.

**Fig. 5:**
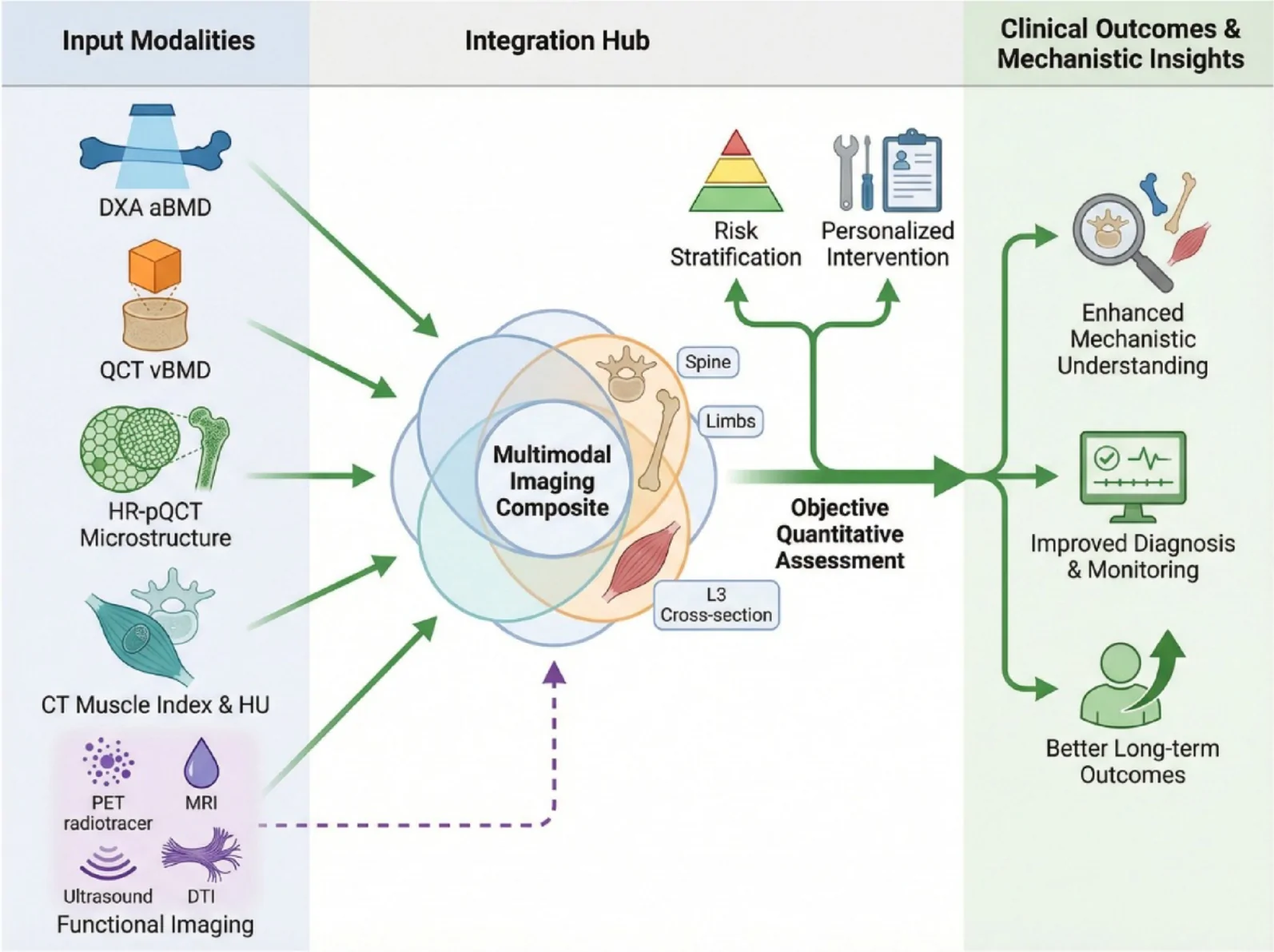
Comprehensive musculoskeletal management for cancer survivors

Despite these promising advances, the field confronts several critical challenges that must be addressed to fully harness the potential of imaging biomarkers. Standardization of measurement protocols across centers is imperative to ensure reproducibility and comparability of data. Additionally, optimizing cost-effectiveness without compromising diagnostic yield remains a practical concern, particularly in resource-constrained settings. The clinical validation of sophisticated biomarkers, especially those derived from functional imaging, requires rigorous prospective studies to establish their predictive value and utility in diverse cancer populations. In this context, the integration of artificial intelligence (AI) and machine learning algorithms holds considerable promise. AI can facilitate automated image analysis, enhance pattern recognition, and integrate multimodal data, thereby overcoming technical bottlenecks and expediting the translation of imaging biomarkers into precision medicine frameworks for cancer survivorship care (Fig. 5).

Looking forward, it is essential to conduct well-designed prospective investigations that focus on treatment-specific and cancer-type-specific imaging biomarker reference standards. Such efforts will enable the development of tailored surveillance strategies and intervention thresholds that reflect the heterogeneity of cancer therapies and patient populations. Furthermore, systematic incorporation of these biomarkers into comprehensive survivorship care pathways will be crucial to enable early detection of musculoskeletal complications, timely therapeutic interventions, and ultimately, improvement in quality of life and long-term survival outcomes. Collaborative multidisciplinary approaches involving oncologists, radiologists, rehabilitation specialists, and data scientists will be key to achieving these goals.

In summary, the evolution of imaging biomarkers in assessing cancer treatment-related bone and muscle sequelae marks a transformative advancement in survivorship medicine. Balancing diverse research perspectives—including structural assessments, functional insights, technical innovation, and clinical applicability—will be vital to realize their full potential. Through continued research, technological innovation, and clinical integration, imaging biomarkers stand poised to become central to personalized management strategies that mitigate musculoskeletal morbidity and enhance the health trajectory of cancer survivors worldwide.

## Data Availability

No new data were created or analyzed in this study. Data sharing is not applicable to this article.
